# Supplementary material for: Chromothripsis during telomere crisis is independent of NHEJ, and consistent with a replicative origin
Source: Genome Res. 2019 May;29(5):737–49. doi: 10.1101/gr.240705.118 (PMC6499312; doi:10.1101/gr.240705.118)
Supplement: Supplemental Material [file supp_gr.240705.118_Supplemental_file_1.zip › contigs/annotated_contigs/DB83/contig.3.DB83_length_607_mean_cov_7.41350906096.docx]

**DB83_length_607_mean_cov_7.41350906096**

```
AGGCAGGAAAGAAGAAAGAGACCAAGGTGCGAGGAGCGGACAGGGAAAGAGACCTCAACATGACCAGGGATGGGAGGAGAGAACTCCAT
 >chr2:11882676-11882831 - E=1e-81 p=4e-02
TACTTAGAAACAGTGGCTGCTATGCTGGGAAGTGTGGACTGTTACAGTGGGCTCTAATTCCAGGAG|TGCTGGAA|TCTCCAGTGCTAA
                                                                           >chr2:118825
GAAAACAGCTACACAGACAGAATGCTAA|GGAG|TCTCACTCTGTCGCCCAGCTGGAGTGCAGTGGCACGATCTCGGCTTACTGCAAGC
13-11882558 - E=2e-15        >chr2:11881527-11881930 - E=7e-225
TCCGCATCCCAGGTTCACATCATTCTCTTGCCTCAGCCTCCTGACTAGCTGGGACTACAGGCACCCGCCACCGTGCCCGGCTCATTTTT

TGTATTTTTAGTAGAGACAGGGTTTCACTGTGTTAGCCAGGATGTGTATTTTTTTATAGAGACAGGGTTTCGCCATGTTGCCCAGGCTG

GTCTCAAACTCCTGAGCTCGAGTGGCAATGTTTGGAACCAATTTACGCAGTTCTTTTCCTTGCAGTTGCCAAACAATATTATTTATCTA

ATGGTCATAACACCTTAACAGTAATAGAACTACTGAACAGCAGTGTTTACTCTGCTCATGGATGAGGTTTCTAGTTG
```
